# A Review on Reductions in the Stress-Intensity Factor of Cracked Plates Using Bonded Composite Patches

**DOI:** 10.3390/ma15093086

**Published:** 2022-04-24

**Authors:** Abdul Aabid, Meftah Hrairi, Jaffar Syed Mohamed Ali, Tamer Ali Sebaey

**Affiliations:** 1 Department of Engineering Management, College of Engineering, Prince Sultan University, P.O. Box 66833, Riyadh 11586, Saudi Arabia; tsebaey@psu.edu.sa; 2 Department of Mechanical and Aerospace Engineering, International Islamic University Malaysia, P.O. Box 10, Kuala Lumpur 50728, Malaysia; jaffar@iium.edu.my; 3 Mechanical Design and Production Department, Faculty of Engineering, Zagazig University, Zagazig 44523, Egypt

**Keywords:** bonded composite, SIF and fuselage, patch, aerospace

## Abstract

In aerospace engineering applications, lightweight material structures are considered to perform difficult service conditions and afford energy efficiency. Therefore, composite materials have gained popularity due to their light weights and high performances in structural design. Mechanical loads and environmental conditions primarily create damage to structural materials, thus numerous studies have considered the repair of the damaged structure. Bonded composite repairs are generally chosen, as they provide enhanced stress-transfer mechanisms and joint efficiencies with the increased use of advanced composite materials in primary and secondary aircraft structural components. Thus, it is essential to have reliable and repeatable bonded repair procedures to restore damaged structural components. However, composite bonded repairs, especially with primary structures, present several scientific challenges in the current existing repair technologies. In this review, a study has been done on the bonded composite repair of damaged structures with the stress-intensity factor (SIF) as the parameter for defining the extent of failure by composite repair and unrepaired material structures. In this work, various types of repair methods and the techniques used by researchers are critically reviewed, and future opportunities are explored. The present study was limited to the composite and aluminium materials that are common in aerospace applications.

## 1. Introduction

**Figure 1:**
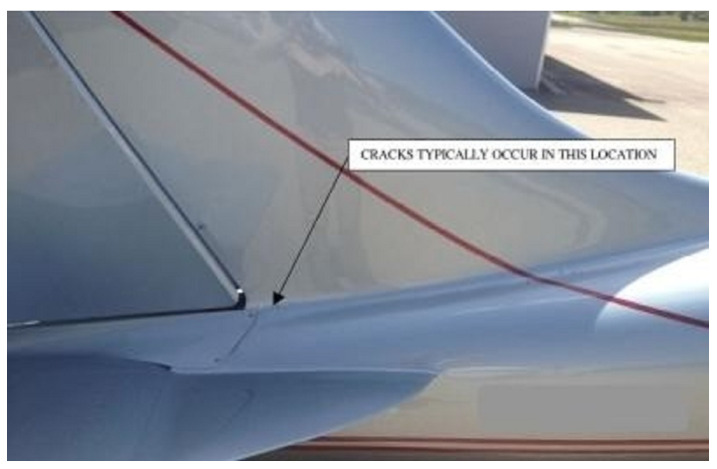
Crack in aircraft body [3]. Reprinted under the Creative Commons (CC) License (CC BY 4.0).

In an aircraft structure, a high amount of maintenance is required to ensure safety, as aircraft bodies can present slight signs that may lead to initiation of a crack, damages, and failure if not replaced with a new structure. Damage types such as delamination, notches, and cracks are inevitable in different fields of engineering application, especially in the aerospace field, and these damages are mostly due to fatigue, corrosion, and accidents. In such studies, a fundamental addition was made by fracture mechanics to the theory of crack propagation of aircraft materials, as well as other important aspects [1,2]. Figure 1 shows a small crack line on a fuselage rear [3] that may lead to a propagating crack due to fatigue. If the damaged material is not widespread, structural repair is the only reasonable solution as replacing the whole component in many cases is not feasible. Passive repairs are usually chosen, as they present improved stress-transfer mechanisms, joint efficiencies, and aerodynamic performance. The objective of this review was to investigate the repair methods using composite patches in research with different techniques, and to identify further modifications/changes for future recommendations. In addition, we observed reductions in the SIF with varied materials, shapes, dimensions and loading conditions in a cracked metallic panel, with effective performance. As an outline of this review, the next section illustrates detailed studies on composite materials and aluminium alloys in aerospace applications. Then, we highlighted the repair methods in engineering applications to simplify a given problem such as analytical, numerical, and experimental. Finally, the last section presents a discussion and recommendations in this field to fill the gaps for the scholar. Several studies have been carried out on cracked structures, and they were repaired using a distinct type of composite-materials patch called a “passive repair” supplied by the aircraft designer.

Fracture mechanics is the study of the mechanical behaviour of a cracked structure subjected to an applied load. A crack is essentially a fracture occurring at the interface of two adjacent layers [4]. The initiated crack can be propagated in three different modes, such as mode-I (opening), mode-II (shearing), and mode-III (tearing). The mechanical behaviour of a solid containing a crack of a specific geometry and size can be predicted by evaluating the SIF for each mode [5]. However, compression stresses in bonded strips tend to buckle, trigger, and accelerate the unstable growth of a crack to the unbonded region of the fractured plate.

Bonded composite repair has been presented as an effective and efficient technique to increase the service life of cracked components. In early attempts, researchers repaired the cracked plate using different types of composite patches [6,7,8,9,10,11,12]. Rabinovitch et al. [13] found that the composite patches were available in numerous types based on the properties of the materials. Moreover, the effects of several patches, such as single or double varieties, in the reduction of the SIF were analysed [14,15,16]. In addition, the patches were designed with different dimensions and shapes to identify their effects on a damaged structure [17]. The effects of an adhesive bonding between the plate and patch were studied and analysed by Ratwani [6] analytically and numerically to determine the stress transmitted from the adhesive to the cracked structure which leads to a decrease in SIF.

Guruprasad et al. [9] numerically estimated the SIF with a composite patch repair on a cracked structure. Later, Naboulsi et al. [10] developed composite patches with a laminate to investigate the repair of a cracked metallic structure. Kam et al. [11] developed an FE model to evaluate the SIF to study the effects of an adhesively bonded composite patch. Mahadesh et al. [12] studied an optimum design of a symmetric (balanced) composite patch on a centre-cracked metallic sheet and identified the SIF. The effects of the adhesive bond, adhesive material, patch size, patch thickness, and patch material in the reduction in the SIF with thermal and structural loading conditions using Cornel University’s FRANC2D/L FE code have been investigated by many researchers [14,17,18,19,20,21,22,23,24,25,26,27,28,29,30,31,32]. Based on the above studies, it can be noted that it is important to know the patch’s dimensions, since the greater thickness of a patch results in a high reduction in the SIF, while on the contrary, the greater thickness also results in a higher weight. Hence, it is desired to keep the thickness of both the patch and the adhesive at the lowest. It was found in the literature that the most common composite patch materials used for the repair of cracked materials are boron–epoxy [33], glass–epoxy, graphite–epoxy [22], E-glass–epoxy [20], carbon–epoxy [21], and carbon-fibre-reinforced plastic (CFRP) [34]. All the above-reported studies were based on mode-I, whereas Maleki et al. [35] repaired an aluminium 2024-T3 plate with a bonded composite patch by evaluating the SIF under mixed-mode conditions. Adhesively bonded joints are increasingly used nowadays in aerospace structural composites due to their convenience and high efficiency in reducing the SIF [36].

## 2. Selection of Material in Aerospace Applications

The material used in aerospace structures should have a high strength-to-weight ratio [37]. We observed that in the last four decades, airplanes manufactured earlier used approximately 80% aluminium alloys. Later, composite materials with a high specific strength and a high specific stiffness gained popularity in engineering applications, especially in aerospace structures, due to an attractive combination of metallic and nonmetallic materials. Thus, currently, the scenario has completely changed, with approximately 80% of aircraft structural parts being made of composite materials.

### 2.1. Aerospace Industry: The Aluminium Alloys

Aluminium material has been used as a primary material for structural components in aircraft since the 1920s, and aluminium alloys are an irresistible choice for the fuselage, wing, and supporting structures of commercial airliners, military cargo planes, and transport aircraft [38]. Developments in the aeronautical industry led to a search for materials with a high strength-to-weight ratio. While this consideration continues to be of first-order importance, a light weight is now necessary, but is not sufficient [39]. The aluminium alloys such as 2024 and 7075, which are extensively used in aircraft structures, are highly susceptible to corrosion [40]. Merati [41] investigated different forms (clad and unclad) of the aluminium alloy 2024-T3, as well the loading directions, thickness, and environment, and identified microstructural features such as particles, grain size, and the clad layer. Aluminium alloy is also available as AA6013-T4, for which the chemical composition was specified in detail by Siqueira et al. [42]. The aluminium AA2014-T6 alloy has been widely utilised in the fabrication of lightweight structures such as aircraft, since it has a high strength--weight ratio and excellent corrosion resistance [37]. Wang et al. [43] investigated the effects of direct cold expansion on the fatigue behaviour of fastening holes in an aluminium AA6016-T6 plate. Aluminium alloys are applicable in aerospace engineering with different series, and some of them are specified in this section. The series are numbered according to the properties of the alloys. Thus, in aerospace engineering applications, aluminium alloys are used as a candidate material to achieve a high strength-to-weight ratio with corrosion resistance.

### 2.2. Aerospace Industry: The Composite Materials

**Figure 2:**
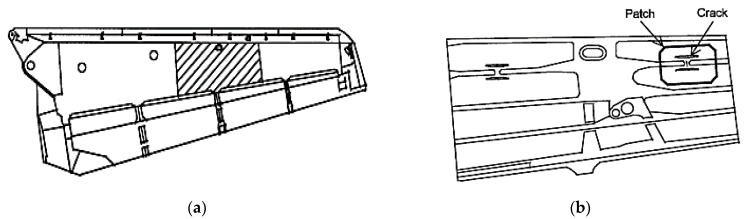
Wing with repair patches: (**a**) lower surface of port wing; (**b**) interior view of the shaded area indicated in (**a**). Reprinted from [46], Copyright 2022, with permission from Elsevier.

A composite material is defined as “a material that consists of two or more constituents which combined at a macroscopic level and insoluble to each other” [44]. Mouritz et al. [45] reviewed the effect of stitching on the in-plane mechanical properties of fibre-reinforced polymer composites, and he showed interest in rapidly growing composite materials in engineering applications. Repairs based on adhesively bonded fibre-composite patches are more structurally efficient and less damaging to the cracked structure than standard repairs based on mechanically fastened metallic patches. Figure 2 illustrates the repair, which has prevented crack growth in three years of further service [46].

Fibrous composites are found in aircraft applications from the first flight of the Wright Brothers’ Flyer 1 in 1903 to the plethora of usage now enjoyed by them in military and civil aircraft. Their growing use has risen due to their high specific strengths and stiffnesses when compared to the more conventional materials [47]. Botelho et al. [48] reviewed the importance of a composite material in aerospace applications, and recommended the future use of hybrid composites in various parts of aircraft structures. Modern composite materials with a high strength and stiffness, which are utilised in aircraft structures to a sufficiently great extent, are now on their way to being widely used in the primary airframe structures of commercial airplanes. We noted in the work of Vasiliev and Razin [49] that the direct substitution of carbon–epoxy composites for traditional wing-stringer stiffened aluminium airframe structures usually resulted in a 10–20% weight reduction. The large-scale use of advanced composites such as carbon fibre in the current programs of development of military fighter aircraft, small and large civil transport aircraft, helicopters, satellites, launch vehicles, and missiles all around the world is perhaps the most glowing example of the utilisation of the potential of such composite materials [50].

Composite materials are also applicable in the possibility of replacing a network of sensors with a wireless network, which is of interest in the monitoring of aerospace structures, as it eliminates wiring and batteries, resulting in reduced bulk and weight, and greatly simplifies the maintenance of the system [51]. Nevertheless, natural fibre-reinforced polymer (NFRP) composites are widely used in automotive and building industries, and there is still room to promote them to use in high-level structural applications such as the primary structural components of aerospace structures [52]. For passive repair, a composite material can play a crucial role in aerospace engineering applications due to its useful properties [53]. However, composite patches are applicable in distinct types, as specified earlier [20,21,22,34], as they are very effective in the reduction of the SIF. Moreover, the shape and size also affect the performance of the repaired cracked structure.

## 3. Methods of Repair

This section describes the three approaches that are used by researchers for bonded composite repair in calculating the SIF. The approaches are: mathematical formulation, numerical simulation, and experimental investigation.

### 3.1. Mathematical Formulation

Ratwani [6] was the first to develop a mathematical model to analyse the crack problem in bonded composite repair. He computed the mode-I SIF using the effects of the cracked surface’s pressure and the body forces. In addition, in his work, a mathematical expression to determine the SIF with and without bonded reinforcement of the cracked structure was developed. Rose [7] repaired a cracked plat using a bonded-reinforcement composite patch. Subsequently, he considered a plate with a semi-infinite crack repaired by using a reinforcing patch bonded to the crack face, and subjected to a uniformly distributed tensile load at the crack [8]. Guruprasad [9] showed that the fracture performance of a cracked plate was substantially improved when providing patches as reinforcement by computing the SIF of a crack through the J-integral method. Naboulsi et al. [10] expressed the SIF equation based on a three-layer technique while considering the cracked plate, adhesive bond, and composite patch. In their work, the fracture-mechanics parameter SIF and the energy-release rate (ERR) of the cracked plate with and without patches were determined. Kam et al. [11] used a modified crack-closure technique to calculate the strain-energy-release rate (SERR) of the cracked structure with the assumption that the strain energy released during the crack extension was equal to the work needed to close the opening crack. Further, they derived the following equation for a centre-cracked plate that related the mode-I total SERR and the SIF for pure bending moments:

(1) $$K_{\psi }=\sqrt{\frac{3{\bar{G}}_{\psi }E_{S}}{t_{s}}}$$

where $K_{\psi }$ is the maximum value of SIF, ${\bar{G}}_{\psi }$ is the total (rotational) strain-energy release rate. Moreover, $E_{S}$ and $t_{s}$ are the Young’s modulus and thickness of the plate, respectively.

Bouiadjra et al. [25] analytically developed a mathematical formula for the determination of the SIF with the effects of the composite patch on a cracked structure of an aluminium sheet with a centre crack. The finite crack length and the dimensions of the patch specified were important parameters in determining the SIF. The effects of the dimensions of the adhesive bond and the composite patch on the SIF is given by following Tada’s equation [14,27,28,29]:

(2) $$K_{\infty }=\sigma_{0}\sqrt{\pi \lambda }$$

where:

(3) $$\pi \lambda =\sqrt{\frac{E_{p}e_{p}}{\beta \left(1+\frac{E_{p}e_{p}}{E_{r}e_{r}}\right)}}$$

and where $\sigma_{0}$ is the uniaxial tensile load and β is the shear-stress transfer length in a representative bonded joint.

Ouinas et al. [30] calculated the SIF for a crack repaired using a composite patch while considering the disbond, and obtained the linear form of the disbonded SIF:

(4) $${\left(K_{\infty }^{d}\right)}^{2}=K_{\infty }^{2}+C_{d}$$

where $K_{\infty }^{d}$ is the SIF at the limit of the composite patch with a disbond d, and $K_{\infty }$ is the SIF without a disbond.

Ahn et al. [54] introduced the p-convergent layered model to propose finite element modelling with a patch repair and a computed SIF based on LEFM and the stress field near the crack tip. Next, a p-convergent high-precision element was developed using the three-dimensional elasticity theory by Ahn et al. [55]; p-convergent ESL (equivalent single layer) element formulation was also used for the two-dimensional response. Ricci et al. [21] calculated the SIF with and without a composite patch using Tada’s formula, and developed an analytical expression using the effects of the cracked plate, adhesive bond, and patch. The expression of SIF was given as:

(5) $$K_{I\left(C\right)}=Y\frac{\sigma_{0}}{\sqrt{k}}\;K_{I\left(C\right)}^{*}=\left(1+\mathrm{BC}\right)K_{I\left(C\right)}$$

in which Equation (5) is the determination of the SIF with a composite patch; and another Equation (5) is a correction factor of a repaired plate for a one-sided repair [6], where BC is the correction term, Y is the geometrical factor, and k represents the spring constant of the given equation for the SIF.

Reddy et al. [34] performed a numerical study of a notch SIF for a centre-cracked plate with a circular hole. They presented a study of the combined behaviours of crack-stop holes and CFRP-reinforced steel plates. Andersson et al. [56] evaluated SIFs by fitting asymptotic displacement functions. Subsequently, the displacements, in general, were more accurately computed than the stresses; this method is, in most situations, preferable compared to stress-based approaches. Talebi et al. [57] investigated the nonlinear fracture mechanics of cracked plates with the effects of adhesive bonding of a composite patch repair. Consequently, the SIF was calculated using the given equation of an infinite plate with a center crack:

(6) $$K_{r}=\sigma_{0}\sqrt{\frac{\pi a\Lambda }{a+\Lambda }}$$

Hattori et al. [58] used the extended-boundary element method (XBEM) formulation to calculate the SIF of a cracked plate composed of an anisotropic material. In addition, Xie et al. (2017) calculated the SIF using a dual-boundary integral equation with a mode-I crack-opening displacement of a three-dimensional cracked plate. Oudad et al. [59] analysed the mode-I crack opening of an aircraft structure panel with a bonded composite patch repair. The SIF of an embedded semielliptical crack in a finite plate subjected to a uniaxial tension load was defined by Ivanez and Braun [16] for single- and double-composite patches. Yu et al. [60] focused on the establishment of the interaction integral (I-integral) for decoupling the force SIFs and coupled SIFs of a crack in functionally graded micropolar material (FGMM). They showed the derivation of the I-integral method from the J-integral method by superimposing an auxiliary field on the actual field. Wang et al. [61] studied the SIFs of double-edged cracked steel plates strengthened with fibre-reinforced polymer (FRP) plates, and used the theoretical expression by Tada to determine the SIF of the bonded-composite repair method.

Based on the existing work, we observed that theoretical and analytical methods were found to be effective approaches in evaluating the SIF of thin-plate structures. Most of the SIFs were found for cracked plates without repairing considering the crack-closer method, Rose’s analytical approach, VCCT, superposition method, and weighted function. Indeed, Rose’s analytical method and the weighted function method were found to be effective in reducing the crack damage, and the results were found to be close to those of the benchmark studies. A further critical analysis is discussed in Section 4.

### 3.2. Numerical Simulation

In this section, we critically review the literature regarding the study of bonded-composite repairs using numerical methods. Numerous software programs have been developed and used in the study of bonded-composite repairs. To compute structural problems, most of the research work over the years has been done using the FRANC2D/L FE code, while in some earlier studies, the MSC NASTRAN FE code was used to validate the existing results. We observed that in recent years, ANSYS and ABAQUS have been the most widely used for simulation and validation purposes.

**Figure 3:**
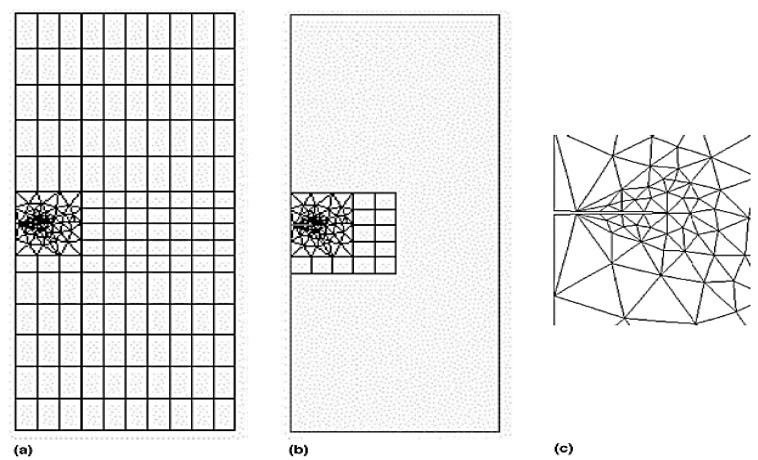
Typical mesh model: (**a**) plate; (**b**) patch; (**c**) close view of crack tip. Reprinted from [26], Copyright 2022, with permission from Elsevier.

Ratwani [6] used the MSC NASTRAN code to validate the analytical results, in which he considered a two-ply, adhesively bonded patch on a centre-cracked aluminium plate with adhesive deboned around the crack area. Naboulsi et al. [10] used four-noded shell elements available in the commercial FE code ABAQUS to model a cracked plate, adhesive bond, and composite patch as a continuum elastic medium for single-sided repair. Kam et al. [11] carried out an independent three-dimensional FE analysis using ABAQUS for a centre-cracked plate with a single-sided patch to validate their experimental results. The FRANC2D/L FE code, which is based on the theory of linear elastic and nonlinear fracture mechanics, was used by many authors to analyse the behaviour of repaired cracks with bonded composite patches in mode-I and mixed-mode [14,17,18,19,20,21,22,23,24,25,26,27,28,29,30,31,32], in which parametric studies were carried out to determine the effects of the crack plate, adhesive bond, and composite-patch parameters on the SIF. In addition, they compared the effects of single-sided bonded-composite and double-side bonded-composite patches. Cornel University’s FRANC2D/L FE code was initially developed without a preprocessor, but later, the CASCA preprocessor, a simple mesh generator, was added to the software, and it recently was modified at the University of Kansas to accommodate multilayers. Figure 3 shows a FRANC2D/L FE code meshing model of a plate and patch.

Bassetti et al. [62] used ABAQUS to perform analyses of the three-layer technique (plate, adhesive, and patch) to investigate the SIF levels, and studied their effects on the SIF due to variations in the composite’s Young’s modulus and adhesive thickness, pretension levels in the patches, and size of the debonded region. Ouinas et al. [63] validated their analytical results using a two-dimensional meshing model in the FRANC2D/L FE code with a semicircular composite patch, and also used a modified virtual crack-closer technique (MVCCI) to estimate the SIF. In this work, the effects of the adhesive thickness, patch thickness, and patch material on the SIF were discussed.

Mohammadi et al. [64] performed three-dimensional FE analyses of the crack growth for a given problem while considering the real crack-front shape of the aluminium centre-cracked plate with the composite patch, and the stress and strain fields of the repaired panels were obtained using the ANSYS FE program. Papadopoulos et al. [65] used the ANSYS 11.0 software to validate their experimental results by using a triangular element, eight-noded SOLID82 element type for a cracked plate, and the same element type was used for an adhesive and composite patch. A plane strain condition was considered in the evaluation of the SIF using the ANSYS FE code. Tsouvalis et al. [66] performed a numerical analysis of a composite patch model using ANSYS 10.0 to validate the experimental results. The SOLID95 element type was used to model the cracked plate, adhesive bond, and carbon/epoxy patch. Oudad et al. [19] used a nonlinear, three-dimensional FE method to compute the contour and the size of the plastic zone ahead of repaired cracks with a bonded composite patch in the ABAQUS FE code.

**Figure 4:**
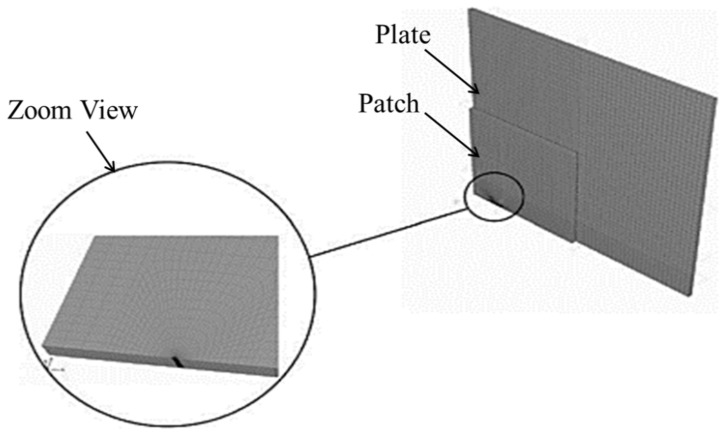
Mesh model of the repaired plate and near the crack tip. Reprinted from [19], Copyright 2022, with permission from Elsevier.

The von Mises criterion was used to determine whether the stress in the materials caused plastic flow, and the Newton–Raphson iterative method was used as an approach to resolve the nonlinear finite element equations. Figure 4 shows the FE meshing model and a close view of the crack tip with a singular element. Next, Bouiadjra et al. [20] used the same method in ABAQUS to simulate the mass-gain estimation of a double patch repair compared to a single patch, and evaluated the reduction in the SIF for single-sided and double-sided composite patches. Lam et al. [67] used the three-layer technique to model a cracked structure using the ABAQUS tool. Eight-node shell elements were used to model the plate with an internal through-thickness crack. Due to the singularity properties of the stress and strain at the crack tip, distorted node elements were used at the crack tip to simulate the stress and strain around the crack tip. Mhamdia et al. [22] evaluated the SIF at a crack front using the virtual-crack-closure technique (VCCT). The VCCT technique uses the principles of linear elastic fracture mechanics (LEFM), and therefore is appropriate for solving problems in which brittle crack propagation occurs along predefined surfaces.

Albedah et al. [15] used ABAQUS to simulate single- and double-sided composite patches with three subsections to model the cracked plate, the adhesive, and the composite patch. The model contained 32,254 eight-node brick elements with 48,381 nodes and a total number of 103,797 degrees of freedom: 17,195 in the plate, 9406 in the adhesive layer, and 7998 in the patch subsections. It presented variations in the SIF according to the crack length for the single and symmetric double patch. Ergun et al. [68] used special quarter-point elements at the crack tip for the calculation of the SIF using the numerical-solution 2D FE code FRANC2D/L. Djamel et al. [69] used the same code to evaluate the SIF using the MVCCI, in which the singularity at the head of the crack was integrated into the solution by replacing the elements at the top of the crack with particular quarter-point elements.

Lei et al. [70] used the higher-order 3D element, eight-noded isotropic SOLID45 to model the substrate panel and adhesive layer and the eight-node anisotropic layered SOLID46 for the composite patch to establish a three-layer model of the repaired panel using ANSYS code. Srilakshmi et al. [71] used ANSYS 13.0 to capture a high-stress gradient near the crack tip. The 20-noded SOLID186 element was used to model the cracked structure, adhesive, and composite patch, which were simulated using the multipoint constrained algorithm (MPC). In the MPC algorithm, all three degrees of freedom were constrained and involved contact and target surfaces that came into contact with one another. In this study, the SIF was estimated from the energy-release rate using VCCT. Benyahia et al. [17,23] considered the effects of the patch shape, dimensions, patch material, and cross-sectional area essential to repair the cracked plate on the reduction in the SIF using ABAQUS. Mhamdia et al. [24] evaluated the SIF using VCCT of the cracked plate with an adhesively bonded composite patch using ABAQUS software. The main contribution was to identify the effects of thermomechanical loading on the cracked plate at different temperatures and tensile loads. Behnam et al. [72] repaired a cracked aluminium panel using the concept of the cohesive-zone model and extended the finite-element method (XFEM) to model the progressive damage in the adhesive of the composite patch repair using ABAQUS FE code.

Reddy et al. [34] numerically investigated a double-notch SIF for a centre-cracked plate with a strengthened crack-stop hole using ANSYS software. Vishnuvardhan et al. [73] studied edge-crack rectangular-plate-bonded composite patches using ANSYS software. To evaluate the reduction in the SIF, they used the SOLID186 element type, which has been used for cracked aluminium plates, and SOLID185 was used for the adhesive bond and composite patch, whereas a CONTACT174 element and TARGET170 elements were also used to make perfect contact between the plate and adhesive, as well as between the adhesive and composite patch. Shinde et al. [74] validated the experimental results through a simulation in ANSYS 15.0 software, using CZM at the interface of the skin and patch. Ivanez and Braun [16] analysed an FE model considering a cracked plate, adhesive bond, and composite patch by using a 3D model generated using the commercial FE code ABAQUS. Talebi and Abedian [57] used ABAQUS to achieve the highest level of stability of crack growth in an aluminium plate in the presence of some constraints, such as weight, load sustainability, and shear stress in the adhesive, and maximum stress in the patch. The crack-growth process was simulated with XFEM under a uniaxial tensile load, and the CZM was used to model the progressive damage in the adhesive of the composite patch repair.

**Figure 5:**
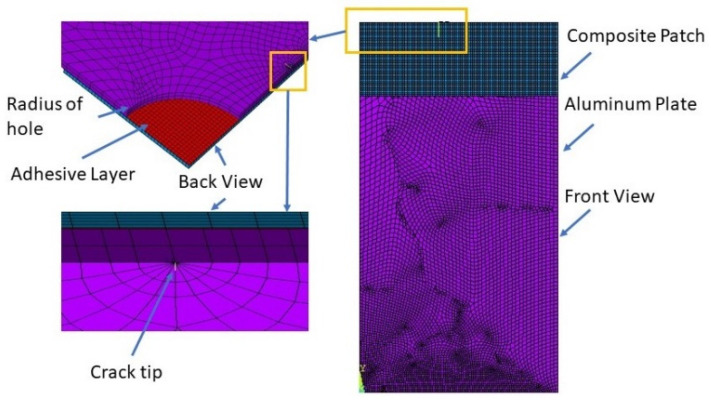
Finite element model and mesh [77]. Reprinted under the Creative Commons (CC) License (CC BY 4.0).

Andersson et al. [56] evaluated the SIF under different loading conditions using low-order displacement with commercial FE codes, and approaches such as XFEM also were employed in an ABAQUS simulation. Maleki and Chakherlou [35] used a flexible-to-flexible contact state for the composite patch and the cracked plate to transfer the load between the contact surfaces of the adhesive layer. Each contact pair consisted of a target element (TARGET 170) and a contact element (CONTACT174). The SIF of the cracked plate was determined in mode-I and mixed-mode. Yu and Kuna [60] examined the influence of material parameters on the force SIFs and couple SIFs for the functionally graded material of a cracked structure (mode-I crack opening) using XFEM. Mahesha et al. [75] used ABAQUS to model the end-notch flexure (ENF) of a specimen in LEFM in mode-I and mixed-mode models. Sadek et al. [76] numerically investigated crack propagation by bonded composite (boron/epoxy) for different patch shapes for repairs of a cracked A5083 aluminium alloy plate. Aabid et al. [77] investigated the SIF of a crack emanating from a hole. The SCF and SIF were evaluated using ANSYS simulation software. Figure 5 shows the corresponding FE model of the stated problem and the mesh that was applied. Aabid et al. [78] investigated the stress-concentration factor (SCF) in an aluminium plate with a hole, and used a composite patch on the hole to study the increase in the life of the material’s structure. They found an approximately 30–40% increase in the life of the material’s structure. Aabid et al. [79] continued the study of SIF determination in a centre-cracked plate and investigated the effects of the parameters using the SIF value of a repaired plate. Baghdadi et al. [80] evaluated the effectiveness of the patch shape on the bonded plate, and they repaired an AI 2024T3 cracked plate using the FE method. Hosseini et al. [81] used a composite patch to investigate fatigue life with the use of the FE method, and the method was also validated using experimental results. Bouzitouna et al. [82] repaired cracked aluminium plates with a hybrid repair technique using a bonded composite patch and drilling a hole in opening mode-I in an elastoplastic analysis.

Benzineb et al. [83] analysed adhesive damage in corroded plates with an angled fracture using various forms and types of patches. As a consequence, the circular patch shape was the best-performing patch shape, and produced intriguing findings throughout this investigation, and the boron/epoxy patch type was also the best. As a structure, a circular pipe was repaired using the same concept; the CFRP patches were designed with an angular shape via the FE method, and the pipe was repaired with a bonded composite patch [84]. Salem et al. [85] presented the effects of different geometric shapes of the composite patches on the damaged area’s variation in the adhesive using corroded and cracked plates. In addition, the mechanical properties of different composites and the thermal loading on the ratio of the damaged area to the crack size were determined using 3D numerical analysis. Lepretre et al. [86] investigated the SIF for repaired damaged plates for only one fracture emanating from a central hole using semiempirical and FE analysis. The SIF at the crack tip was defined and evaluated using VCCT. In addition, the FE findings were compared to experimental data collected by the authors for various reinforcement configurations.

One of the most cost-effective methods used to investigate reductions in the SIF is the numerical approach, and this review provides research studies and their analyses. Based on the current review of the numerical approach, we found numerous studies that have shown a reduction in the SIF using FEM via ANSYS, StarCCM, ABAQUS commercial software’s also fracture analysis code: Franc2D, and Franc3D Cornell Fracture Group. It was observed that meshing was critical to obtaining accurate results, while other effects such as modelling, proper boundary conditions, and a high-speed processor were also important. However, the best results are derived from meshing; therefore, most researchers performed mesh-independence tests and mesh-sensitivity analyses for optimum results, Furthermore, FEM results were validated with experimental work once results were validated by performing parametric studies. Section 4 illustrates the critical analyses of numerical works.

### 3.3. Experimental Works

Experimental outcomes are very significant, as they form the benchmark for validation of analytical methods and numerical simulations. In this section, a review of such experimental work conducted on bonded composite repairs is carried out. The prototype preparation of the specimen for aerospace structures, such as the plate/panel, were tested using the available equipment and facilities for benchmark results to validate the analytical and numerical results.

Anderson et al. [87] experimentally investigated the crack-growth rate for aluminium adherents and two adhesive bonds (AF-55 and AF-127), and a fatigue cycle with a fixed average remote-stress maximum of 108.4 Pa at a stress ratio of 0.1 was applied. R. A. Jurf and R. B. Pipes [88] experimentally investigated the critical SIF and SERR for the interlaminar fracture characteristics of a graphite/epoxy composite material (AS1/3501-6) under opening, shearing, and mixed-mode conditions. Ravi-Chandar and Knauss [89] examined the effects of stress waves on the behaviour of running cracks; in particular, with the influence of stress waves on the branching phenomenon, as well as the crack curving under transient stress waves. Baker [90] experimentally determined the fatigue-crack propagation in cracked aluminium alloy (2024-T3) panels repaired with boron/epoxy composite patches, and adhesively bonded with either an epoxy nitrile film adhesive or an acrylic adhesive. Seo and Lee [91] investigated the fatigue-crack growth behaviour of thick panels repaired with a bonded composite patch using the SIF range and the fatigue-crack growth rate. They determined the SIF of patched cracks from experimental results by comparing the crack-growth behaviour of specimens with and without a repair.

**Figure 6:**
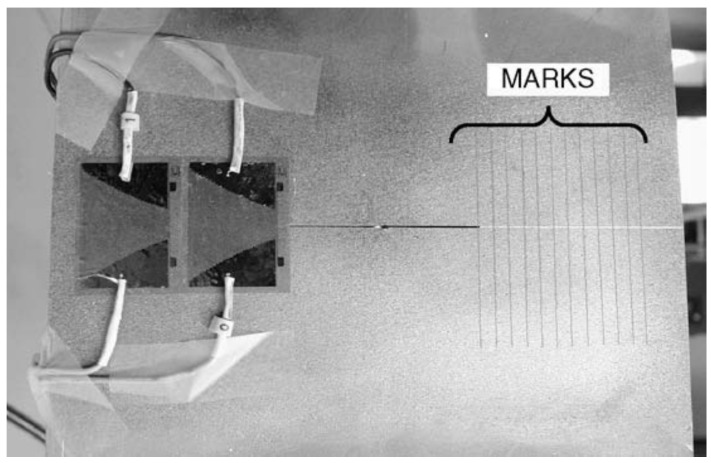
Crack gauges and optics. Reprinted from [66], Copyright 2022, with permission from Elsevier.

Hosseini-Toudeshky et al. [64] carried out an experimental study on the fatigue-crack growth in mode-I for an aluminium panel (2024-T3) with a centre crack and repaired with a graphite/epoxy composite patch. They prepared the specimen according to the ASTM standard E-647 specification; it was then bonded with a unidirectional composite patch with different layers and tested in a tensile test machine. Papadopoulos et al. [65] experimentally evaluated the SIF for an edge-cracked plate with a bonded composite patch subjected to a uniaxial tension load in which the specimen was composed of Lexan (PCBA) with Plexiglas (PMMA) patches. Tsouvalis et al. [66] prepared a centre-cracked plate specimen with cracked gauges and made optical measurement marks, as shown in Figure 6, to identify the crack-growth rate. They monitored the crack length based on two techniques: in the first, special crack gauges were applied at one of the crack tips; and in the second, for the other crack tip, optical measurements were taken. The specimen was composed of steel bonded with a carbon/epoxy composite patch.

Lam et al. [67] repaired a steel plate with a single-sided bonded composite patch composed of CFRP and evaluated the SIF of the cracked structure. A total of six tension tests, using five specimens with CFRP patching and one specimen without patching, were conducted to study the effects of the CFRP patching on the strain distribution through the cracked steel plate. All the steel plate specimens (CAN/CSA-G40.21 300W) had outside dimensions of 6.35 mm thick, 400 mm wide, and 750 mm long. Ricci et al. [21] compared analytical and numerical results with experimental work using a cracked plate (aluminium alloy) bonded with a composite patch (carbon/epoxy) using an adhesive layer (Loctite Hysol EA95, Cytec FM73, and AF163-2K) under different loading conditions. The experimental tests’ outcomes demonstrated a reduction in the average stress of about 30% due to the patch, which translated into an increase of about 50% in the fatigue life.

**Figure 7:**
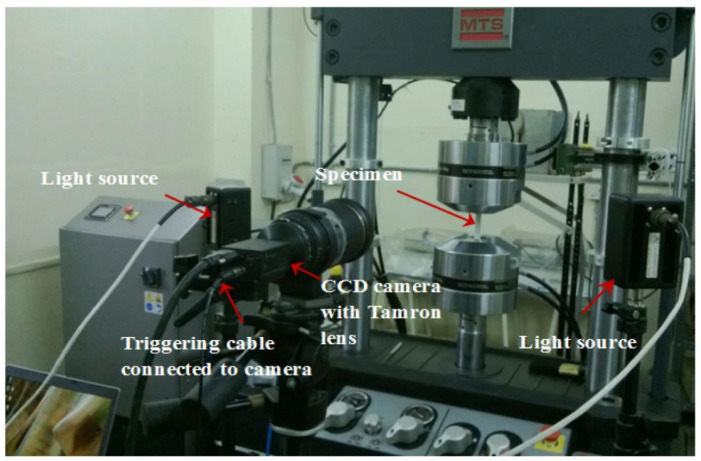
Experimental setup of 3D-DIC. Reprinted from [71], Copyright 2022, with permission from Elsevier.

Srilakshmi and Ramji [71] repaired an aluminium 2014-T6 panel bonded with a CFRP composite patch using Araldite 2011 adhesive bond. A digital image correlation (DIC) set up along with the loading equipment was used to investigate the strain values. The 3D-DIC system comprised a pair of CCD cameras used to capture images of the specimen. The specimens were loaded using a computer-controlled MTS Landmark servohydraulic cyclic testing machine with a 100 kN capacity, as shown in Figure 7.

**Figure 8:**
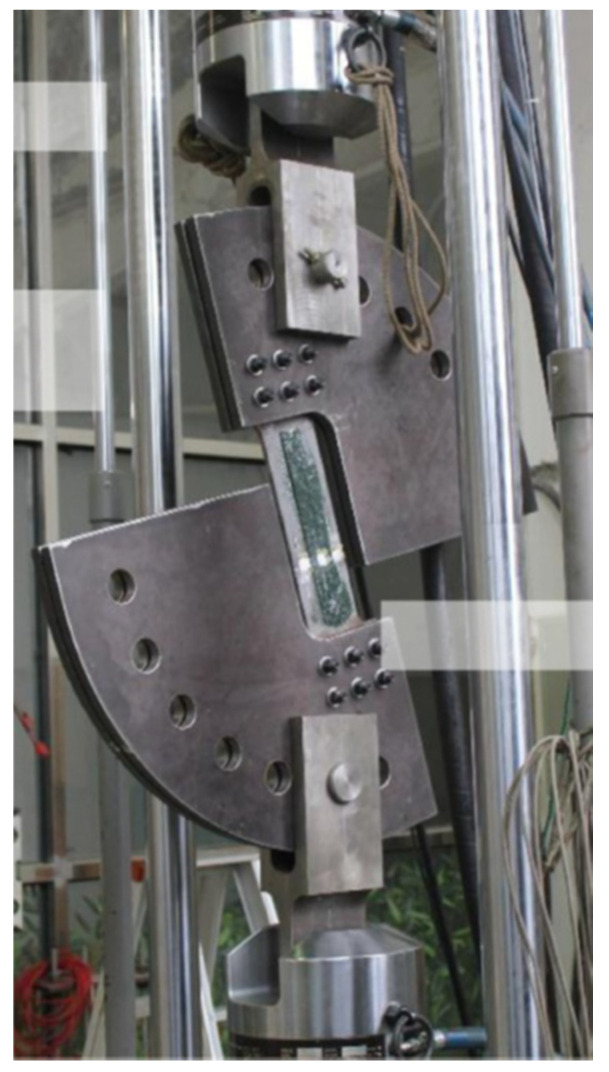
The 100% mode-I fixture and specimen configuration. Reprinted from [92], Copyright 2022, with permission from Elsevier.

Maleki and Chakherlou [35] used a Zwick static tensile testing machine to find the fracture strength of a given specimen. The specimen was a 2024-T3 aluminium alloy plate with an initial U-notch generated at the middle of the specimen’s edge by a milling machine. Then, the specimen was subjected to cyclic loading in a fatigue-testing machine. Two batches of specimens, including a simple edge crack and repaired batches, were evaluated at three different values of composite patch thickness and three different values of adhesive thickness under different loading levels (100%, 75%, 50%, 25%, and 0% in mode-I). Figure 8 shows the experimental setup for the mode-I condition.

Zarrinzadeh et al. [93] experimentally repaired pipes composed of the aluminium alloy 6063 with a composite patch and tested the growth behaviour of the fatigue crack using a fatigue machine. They evaluated the SIF for an inclined crack in a pipe under a uniaxial tensile load. The crack was made in the pipe using the electrical discharge machining (EDM) technique instead of the conventional wire-cutting method. Seriari et al. [94] investigated the fatigue-crack growth of an edge-cracked aluminium alloy plate (2024 T351) that was repaired using a bonded composite patch (boron/epoxy) under constant and variable-amplitude loading (VAL). In addition to constant-amplitude loading (CAL), the effects of a single overload and a band overload were investigated using repaired and unrepaired plates. Albedah et al. [95] experimentally investigated the effects of adhesive disbond and thermal residual stresses on the fatigue life of cracked 2024-T3 aluminium panels repaired with a carbon/epoxy composite patch. Hart and Bruck [96] experimentally investigated the characterization and modelling of a low-modulus 5052-H32 aluminium centre-cracked tension specimen patched with an E-glass epoxy composite by using digital image correlation (DIC) surface displacements. Chen et al. [92] experimentally studied the mixed-mode fatigue behaviour of centre-cracked steel plates repaired with CFRP materials and Araldite 420 adhesive bond.

Apart from the SIF, the fatigue life of fractured 2024-T3 aluminium [97,98,99] and 7075-T6 [99] alloy mended with a bonded composite patch was investigated after a single overload. The load history, bonded patch repair procedure, and overload application were all given special care. Under constant amplitude loading, fatigue tests were performed on V-notched repaired and unrepaired specimens that were naturally precracked. Then, a fractographic analysis was performed on the failed specimens to examine the fatigue findings. It was discovered that if the fracture was fixed before the overload was applied, the overload’s retardation impact was reduced by the patch. In addition, to assess the plasticity generated by the overload on the patched plate and to study the adhesive damage after this overload was applied, the elastic-plastic finite-element technique was utilised [85]. Fatigue experiments were carried out on V-notch cracked specimens utilising two aluminium alloys (2024-T3 and 7075-T6) that were repaired with bonded carbon/epoxy patches under continuous amplitude loading with a positive stress ratio. Rectangular, trapezoidal, and triangular composite patch forms were employed. The effects of patch orientation on the repair efficiency were studied using left- and right-oriented triangular patch designs [100].

Regarding experimental works, we found very few studies that were recognised as compared to those that used the FEM. Most of the studies only determined the SIF for damaged structures, and only a few works were found that determined the SIF for repaired structures. In experimental works, strain gauges were used to determine the order to define the SIF value using the below formula:

(7) $$2G\varepsilon_{x^{\prime }x^{\prime }}=\frac{1}{\sqrt{r}}\left[\frac{K_{1(\mathrm{total})}}{\sqrt{2\pi }}\left(k\cos\frac{\theta }{2}-\frac{1}{2}\sin\theta \sin\frac{3\theta }{2}\cos2\alpha +\frac{1}{2}\sin\theta \cos\frac{3\theta }{2}\sin2\alpha \right)\right]$$

where k is the bulk modulus of a cracked aluminium specimen, which is given by the equation; *G* is the shear modulus of the cracked aluminium specimen; *ε*_aa_ is the measured strain along the radial line from the crack front; r is the radial location of the strain gauge from the crack front; and θ and α are the strain gauge’s angular location and orientation, respectively.

To determine the SIF, some studies used the digital image correlation method and found it to be effective. However, no studies were recognised in which DIC was used for the repaired plates; this review presented guidelines for researchers when utilising this method on repaired structures. A further critical analysis of the review is given in Section 4.

## 4. Discussion and Future Recommendations

Katnam et al. [101] reviewed several scientific challenges and opportunities to develop cost-effective and certifiable technologies for composite bonded repairs. As damage mechanisms in aluminium are often very complicated due to their inherent homogenous and isotropic material behaviours, nondestructive inspection poses several challenges to accurate and reliable damage assessments. Radaj [102] reviewed extended SIF concepts for describing the stress field at the pointed crack or slit tips for different types of aerospace structures.

**Figure 9:**
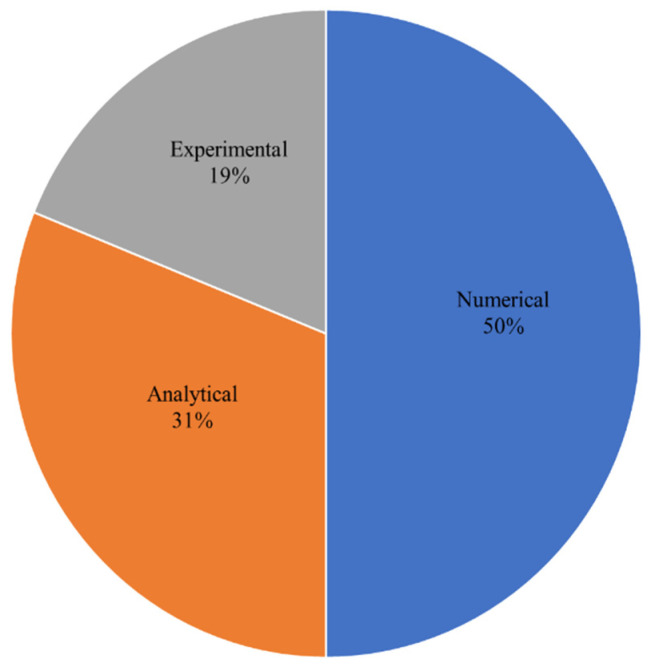
Solution methods.

A structure can be damaged by low-energy effects, and cracks initiated in its inner layer may propagate and cause the failure of the whole structure. These types of damages will lead to significant reductions in the mechanical properties of the material. For the repair of such damaged isotropic plates, composite material patches have been widely used, and have proven useful. After an in-depth and exhaustive review of previous works on the passive repairs of structures, we found that the work was done based on three methods: analytical, numerical, and experimental, as shown in Figure 9.

**Figure 10:**
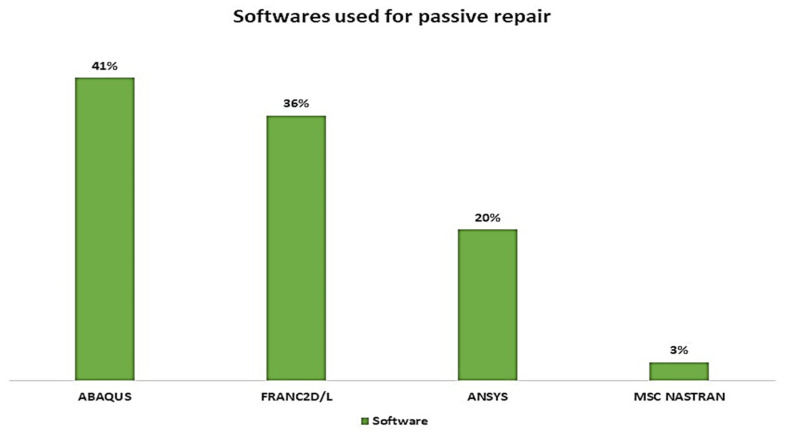
Software programs used for modelling passive repairs.

Excellent work has been reported using numerical methods while considering the linear elastic fracture mechanics (LEFM) method and extended FE (XFEM) method. We also observed that there are numerous software programs available to evaluate the SIF, and most of the work done was by using ABAQUS and FRANC2D/L software. Initially, the FRANC2D/L FE code could only solve simple two-dimensional FE problems; later, Cornell University developed a new version of this software, the FRANC3D/L FE code, which can solve three-dimensional FE problems, but it has not been widely used, as many researchers opted for the ANSYS software, which is versatile in modelling and analysis due to an advanced pre-processor and post-processors. Figure 10 shows the usage of particular software programs (in percentages) to calculate the SIF for a cracked structure bonded with composite patches during the last four decades (1979 to 2022).

Mathematical modelling of a cracked structure was studied by some researchers [6,7,8,11,30,34,54,59,60] with and without composite patches. In the literature, we observed that there were many derivative methods used to calculate the SIF of cracked materials, such as ERRs and the VCCT method. Furthermore, some experimental works are yet to be explored and there are some experimental procedures that are not being used to validate existing results. It was found in the literature that for repaired and unrepaired cracked structures, to determine the SIF, only 19% of the work has been done experimentally (including fatigue-crack growth behaviour) compared to numerical and analytical solutions in the last four decades.

We found that approximately 60% of the work was done in mode-I compared to mode-II and mode-III (or mixed-mode). Meanwhile, researchers can also examine the repair of the shearing mode and tearing mode with bonded composite material patches. We noted that the effects of patch size, patch thickness, patch material, and several applied patches on the SIF need to be studied, as they had a substantial impact on mode-I results. We also observed that most of the work was performed on edge-cracked plates compared to centre-cracked plates in bonded composite repair. Subsequently, it is a signed mark for researchers to look at the repair of the centre-cracked plate. The use of the composite material is now open with new advanced technology and several types of composites as specified in section one is used for different engineering application, especially for aerospace structure.

Table 1 illustrates some of the latest works that can be used to fill the gaps in research on repairing damaged aircraft structures with different types of composite materials and using newly developed techniques and software tools. However, some of the composite materials are yet to be used for passive repairs in order to identify their performance in damaged aerospace structures. Furthermore, functionally graded materials (FGM) have recently gained popularity in structural enhancement in engineering applications, and these materials can also be used for passive repair of damaged structures.

Structural bonded repairs need to be precisely analysed and designed to re-establish damaged components. Structural repairs should maximise the repair’s effectiveness and minimise the risk of material failure under service conditions. However, the performance of bonded patches depends on numerous factors, such as processing, materials, and geometrical constraints, and thus it may be expected that the patch behaviour will be complicated. Advanced computational modelling techniques (e.g., damage/fracture mechanics and statistical methods) could offer accurate numerical solutions for reliable and optimised repair. Human errors and inconsistencies in repair processes can significantly influence the structural strength and durability of bonded composite repairs.

## 5. Conclusions

In this work, different methods to determine the SIF for a cracked structure were reviewed. Subsequently, a study was made of the composite materials used for the repair of cracked structures, and we found that the most widely used composite material was boron/epoxy due to its attractive combination of properties. Numerous works on the repair of plate, pipe, and shell structures using a composite patch have been reported in the last four decades. Different techniques were employed to study the effects of composite patches on the SIF, and we found an approximately 55% to 65% reduction in the SIF for single-sided patches and an 80% reduction in the SIF for double-sided patches after repair of the cracked plate in mode-I and mixed-mode loads. In addition, we found that adhesively bonded repairs were adequate to transmit stresses, and the correct selection of the adhesive was vital in the manufacturing of engineering structures. In this review, guidelines were established for researchers who seek to use composite patches in aerospace engineering applications. These guidelines regard the types of composite materials selected and the repair methods for a cracked structure. Moreover, the discussion and critical review can offer scholars a wide vision and suggestions for further exploration of gaps that must be closed by future research.

## Figures and Tables

**Table 1:** Different methodologies used for cracked-plate and delamination control.

Type of Structure	Technique Adopted	Number/Types of Composite Patches	Focused Parameters	Opportunity for Further Research	Reference
Aluminium plate	LEFM and ANSYS simulation	Composite patch with piezoelectric actuator	SIF repaired and unrepaired plate	Experimental investigation for validation of numerical results	[103]
Aluminium plate	Design of experiments	Single-sided composite patch	The optimum solution for SIF reduction	Further continued with a double-sided composite patch	[33]
Aluminium plate	ANSYS simulation	Composite patch with piezoelectric actuator	Effect of patch on SCF reduction	Experimental investigation for validation of numerical results	[77]
Aluminium plate	J integral/FEM	1 (carbon/epoxy patch)	fatigue-crack propagation	The dimension of the patch can be also redefined	[95]
Aluminium plate	von Mises stress, J integral/FE model (ABAQUS)	1 (boron/epoxy patch)	Fatigue life/failure criteria/crack tip under plastic zone	SIF can be calculated in this case	[59]
Superalloy (novel model)	High- and low-cycle fatigue (CCF) loading	No patch	Crack-closure effect and Crack-growth behaviour	Effect of using composite patches on SIF	[104]
32 layers of carbon-fibre-reinforced epoxy plate	Strain energy release rate (G) for stress-ratio effect	No patch	Mixed-mode fatigue delamination growth/damage mechanisms/crack- growth rate	Delamination control	[105]
Aluminium plate	Step heating thermography/ANSYS simulation	1 (carbon/epoxy patch)	Evaluated the effects of defect type (delamination and disbond)/heat transfer	Evaluate SIF	[106]

## Data Availability

Not applicable.
